# Comparison of homologous and heterologous prime-boost vaccine approaches using Modified Vaccinia Ankara and soluble protein to induce neutralizing antibodies by the human cytomegalovirus pentamer complex in mice

**DOI:** 10.1371/journal.pone.0183377

**Published:** 2017-08-16

**Authors:** Flavia Chiuppesi, Felix Wussow, Louise Scharf, Heidi Contreras, Han Gao, Zhuo Meng, Jenny Nguyen, Peter A. Barry, Pamela J. Bjorkman, Don J. Diamond

**Affiliations:** 1 Department of Experimental Therapeutics, Beckman Research Institute of the City of Hope, Duarte, CA, United States of America; 2 Division of Biology and Biological Engineering, California Institute of Technology, Pasadena, CA, United States of America; 3 Center for Comparative Medicine, California National Primate Research Center, Department of Pathology and Laboratory Medicine, University of California Davis, Davis, CA, United States of America; University of St Andrews, UNITED KINGDOM

## Abstract

Since neutralizing antibodies (NAb) targeting the human cytomegalovirus (HCMV) pentamer complex (PC) potently block HCMV host cell entry, anti-PC NAb induction is thought to be important for a vaccine formulation to prevent HCMV infection. By developing a vaccine strategy based on soluble PC protein and using a previously generated Modified Vaccinia Ankara vector co-expressing all five PC subunits (MVA-PC), we compared HCMV NAb induction by homologous immunization using prime-boost vaccine regimen employing only PC protein or MVA-PC and heterologous immunization using prime-boost combinations of PC protein and MVA-PC. Utilizing a recently isolated anti-PC NAb, we produced highly pure soluble PC protein that displayed conformational and linear neutralizing epitopes, interfered with HCMV entry, and was recognized by antibodies induced by HCMV during natural infection. Mice vaccinated by different immunization routes with the purified PC protein in combination with a clinically approved adjuvant formulation elicited high-titer and durable HCMV NAb. While MVA-PC and soluble PC protein either alone or in combination elicited robust HCMV NAb, significantly different potencies of these vaccine approaches were observed in dependence on immunization schedule. Using only two immunizations, vaccination with MVA-PC alone or prime-boost combinations of MVA-PC and PC protein was significantly more effective in stimulating HCMV NAb than immunization with PC protein alone. In contrast, with three immunizations, NAb induced by soluble PC protein either alone or combined with two boosts of MVA-PC increased to levels that exceeded NAb titer stimulated by MVA-PC alone. These results provide insights into the potency of soluble protein and MVA to elicit NAb by the HCMV PC via homologous and heterologous prime-boost immunization, which may contribute to develop clinically deployable vaccine strategies to prevent HCMV infection.

## Introduction

Since human cytomegalovirus (HCMV) is a leading cause of birth defects in newborns and complications in immunologically vulnerable individuals such as transplant recipients and AIDS patients, developing a vaccine to prevent HCMV infection is considered a major public health priority [1, 2]. Yet, an HCMV vaccine candidate that would be considered for licensure remains elusive [3]. The absence of HCMV animal models, poorly understood immune correlates of protection, incomplete protection by naturally acquired immunity, and difficulties of conducting sufficiently powered vaccine trials are a number of obstacles that complicate HCMV vaccine development [3, 4]. Despite these impediments, promising findings for the feasibility of an effective HCMV vaccine candidate have been obtained with an approach based on envelope glycoprotein gB combined with MF59 adjuvant, showing partial efficacy to prevent primary infection of young women or adolescent girls and ability to reduce viremia in solid organ transplant recipients [5–7]. While the protective mechanisms of gB/MF59 remain largely unknown, the gB/MF59 clinical trials may suggest that a vaccine strategy with enhanced ability to stimulate virus-specific immunity could significantly alter the outcome of HCMV infection and disease.

As neutralizing antibodies (NAb) interfere *in vitro* with glycoprotein-mediated virus entry into host cells, NAb are thought to contribute to the prevention of HCMV infection [8, 9]. Yet, *in vivo* data that supports this conclusion remain relatively sparse [10–12]. Early studies based on inhibition of fibroblast (FB) entry by FB-adapted HCMV laboratory strains such as AD169 and Towne identified gB as a major NAb target [13]. In recent years, however, it has been recognized by studies using more “clinical-like” HCMV strains including TB40/E, TR, and VR1814 with intact cell tropism that NAb responses blocking HCMV entry into many non-FB cell types target in majority a pentamer complex (PC) composed of gH, gL, UL128, UL130, and UL131A [14–16]. While this complex is dispensable for FB entry, it is required for virus entry into epithelial cells (EC), endothelial cells, monocytes/macrophages, and other cell types considered important for virus dissemination and transmission [14, 17–19]. In contrast to NAb targeting gB, gH, or gO epitopes that broadly interfere with HCMV host cell entry, NAb recognizing predominantly conformational epitopes of the UL128/130/131A subunits of the PC are highly potent in preventing HCMV entry into cell types such as EC, though they are unable to prevent FB infection [16, 20, 21]. In addition, we have shown that PC-specific NAb potently interfere with HCMV entry into primary placental cytotrophoblasts, indicating that these antibodies could be important to block vertical transmission [21]. These findings suggest that induction of PC-specific NAb could be particularly important for a vaccine candidate to prevent congenital HCMV infection.

As a result of these discoveries, we and others have developed promising vaccine strategies to augment HCMV NAb induction in different animals models by eliciting immunity targeting the PC [22–25]. Most of these strategies enabling enhanced HCMV NAb induction employed subunit vaccine approaches based on recombinant protein or viral vectors, which avoids or reduces safety concerns and issues of production that are potentially associated with vaccine approaches based on live-attenuated or replication-defective HCMV strains or other approaches utilizing whole HCMV virion components. We recently developed a vaccine approach to elicit high-titer and durable HCMV NAb in mice and rhesus macaques by a Modified Vaccinia Ankara vector (MVA) co-expressing all five PC subunits, referred to as MVA-PC. In this study, we developed a vaccine strategy based on soluble PC protein and compared HCMV NAb induction in mice by homologous and heterologous prime-boost vaccine approaches using soluble PC protein and MVA-PC. Our results highlight the potency of these subunit vaccine approaches to stimulate high-titer NAb responses by the HCMV PC dependent upon prime-boost immunizations schedule.

## Materials and methods

### Cells and viruses

ARPE-19, MRC-5, and baby hamster kidney cells (BHK-21) (American Type Culture Collection [ATCC]) were maintained and propagated by standard procedures. HCMV TB40/E expressing GFP was derived from TB40/Ewt-GFP BAC DNA, a kind gift from T. A. Shenk and E. A. Murphy (Princeton University, NJ) [26]. The construction and generation of MVA-PC was described previously [27]. HCMV strain TB40/E and MVA-PC virus stocks were prepared as described previously following virus propagation of ARPE-19 or BHK-21 cells, respectively [21, 27, 28].

### Protein expression and purification

Soluble PC protein was produced using HEK 293-6E cells (NRC-BRI, Montreal, Canada) following co-transfection of five plasmids encoding the individual PC subunit genes within a pTT5 vector (NCR-BRI). HCMV gH was truncated at 714 amino acids, adding a C-terminal 6xHis tag. The signal peptide from each subunit was replaced with HIV *gp120* signal peptide [29]. Cells were transfected according to manufacturer protocol [30] and supernatant collected 120 hours later. Secreted PC was captured on Ni^2+^-NTA resin (GE Healthcare, Pittsburgh, PA) and the eluate was further purified using a proprietary monoclonal antibody recognizing a conformational epitope on UL128/130/131A (12E2, [21]) bound to a HiTrap NHS-Activated HP sepharose column (GE Healthcare). After capturing, PC protein was eluted with 3M MgCl_2_ then immediately performed a buffer exchange to 20 mM Tris pH 8.0, 150 mM NaCl followed by gel filtration using a GE Superdex 200 10/300G column. Protein concentration was assessed via Bradford assay and presence of all five subunits was verified via SDS-PAGE followed by Coomassie staining analysis.

### Antibody depletion

Microtiter wells (Costar) were coated overnight with purified PC (5 μg/ml) in bicarbonate/carbonate buffer (100 mM, pH 9.6). Wells were blocked with 1% BSA/PBS. Intravenous immunoglobulins (IvIg, Privigen, CSL Behring, Marburg, Germany) were diluted to 200 μg/ml in 0.5% BSA/PBS. HCMV seropositive (Lots BM204360 [Seracare 60] and BM204371 [Seracare 71]) and negative (Lot BM216642 [Seracare neg]) sera (SeraCare Life Sciences, Oceanside, CA) were diluted 1:50 in 0.5% BSA/PBS to approximate an IgG concentration of 200 μg/ml. To deplete PC-specific antibodies, IvIg and sera were serially incubated five times for one hour in the coated wells. Mock depletion was performed using wells coated with bicarbonate/carbonate buffer and blocked with 1% BSA/PBS.

### ELISA

Microtiter wells (Costar) were coated overnight with proprietary anti-gH monoclonal antibody 21E9 [21] (1 μg/ml) diluted in bicarbonate/carbonate buffer (100 mM, pH 9.6). Wells were blocked with 5% skim milk and incubated with 2 μg/ml purified PC protein in PBS. In-house developed mouse monoclonal antibodies (1B2, 54E11, 21F6, 12E2, 13B5, 18F10, 62–11, 2–80 [21]) and anti-gH AP86 [31] were biotinylated using EZ-Link NHS-PEG4-biotin biotinylation kit (Thermo) following manufacturer’s instructions and added to the wells at a concentration of 10 μg/ml. Streptavidin-HRP was used at a dilution of 1:3000. Wells were developed with 3,3',5,5'-tetramethylbenzidine (Thermo). Absorbance at 450 nm was read using FilterMax F3 (Molecular Devices, Sunnyvale, CA). For the analysis of PC-specific human serum antibodies, the same protocol described above was used to coat the wells with the purified PC. Depleted and mock-depleted samples were 2-fold serially diluted in 2.5% skim milk starting from a dilution of 50 μg/ml (or 1:200 for serum samples) to 0.4 μg/ml (or 1:25600 for serum samples). Anti-human IgG horseradish peroxidase conjugate (Promega, Madison, WI) was used 1:2000. For the analysis of PC-specific binding antibodies in mouse serum, the same protocol was used but serum samples were diluted four-fold from 1:800 to 1:204,800. Anti-mouse IgG horseradish peroxidase conjugate (Sigma-Aldrich, St. Louis, MO) was used at a dilution of 1:2,000. Half-maximal binding of antibodies (EC50) was derived by plotting absorbance at 450 nm as a function of the logarithm of serum dilution to obtain a sigmoidal curve analyzed using the four-parameter logistic (4PL) nonlinear regression model (Prism 7; GraphPad Software, San Diego, CA).

### Mouse immunization

The IACUC of the Beckman Research Institute of City of Hope approved protocol #98004 assigned for this study. All study procedures were carried out in strict accordance with the recommendations in the “Guide for the Care and Use of Laboratory Animals of the National Institutes of Health”. BALB/c mice (Jackson Laboratory, Bar Harbor, ME) were intramuscularly (i.m.), intraperitoneally (i.p.), or subcutaneously (s.c.) immunized three times four weeks apart with 1 μg of purified PC admixed with a squalene-based oil-in-water nano-emulsion (AddaVax, InvivoGen, San Diego, CA). In the prime-boost experiment, groups of five BALB/c mice were primed with MVA-PC (5x10^7^ PFU, i.p.) or purified PC (1 μg admixed with AddaVax, i.p.), and boosted one or two times with the same dose of purified PC or MVA-PC, respectively. A third and fourth group of animals received two or three immunizations with MVA-PC or purified PC alone following the same schedule. Mice were bled at week 0, 3, 7, 11 and at various time points afterwards.

### Neutralization assay

For the inhibition of HCMV entry study, a neutralization assay was performed as previously described [21, 25, 28]. Briefly, ARPE-19 cells were seeded at 1.5x10^4^ cells/well in a clear-bottom 96-well plate (Corning). Approximately 24 h later, the medium in every plate was replaced with 50 μl per well of fresh growth medium. Purified PC, NAb 62–11 and 1B2 [21] were 2-fold serially diluted starting from 300 μg/ml, 100 μg/ml and 2 μg/ml respectively. PC and NAb dilutions were mixed with complete growth medium containing approximately 9,000 PFU of HCMV TB40/E and incubated for 2h at 37°C. The mixture was transferred to the cells in duplicate wells. After 48 hours, cells were fixed and IE-1 immunostaining performed using Vectastain ABC kit (Vector Laboratories, Burlingame, CA) according to the manufacturer's instructions. NAb concentration inhibiting 50% of the virus infectivity (IC50) was also calculated as previously described [21]. For the analysis of the HCMV-specific NAb response in immunized mice, the same protocol was used. Mouse serum starting dilution was 1:50. For the evaluation of HCMV NAb in human serum products, depleted and mock-depleted IvIg starting dilution was 50 μg/ml, while depleted and mock-depleted human serum dilution started from 1:200.

### Statistical analysis

GraphPad Prism software version 7 (GraphPad Software Inc., San Diego, CA) was used to compare NAb and binding antibody titers between vaccine groups by statistical analysis using multiple t-test.

## Results

### Production and characterization of soluble HCMV PC protein

**Fig 1:**
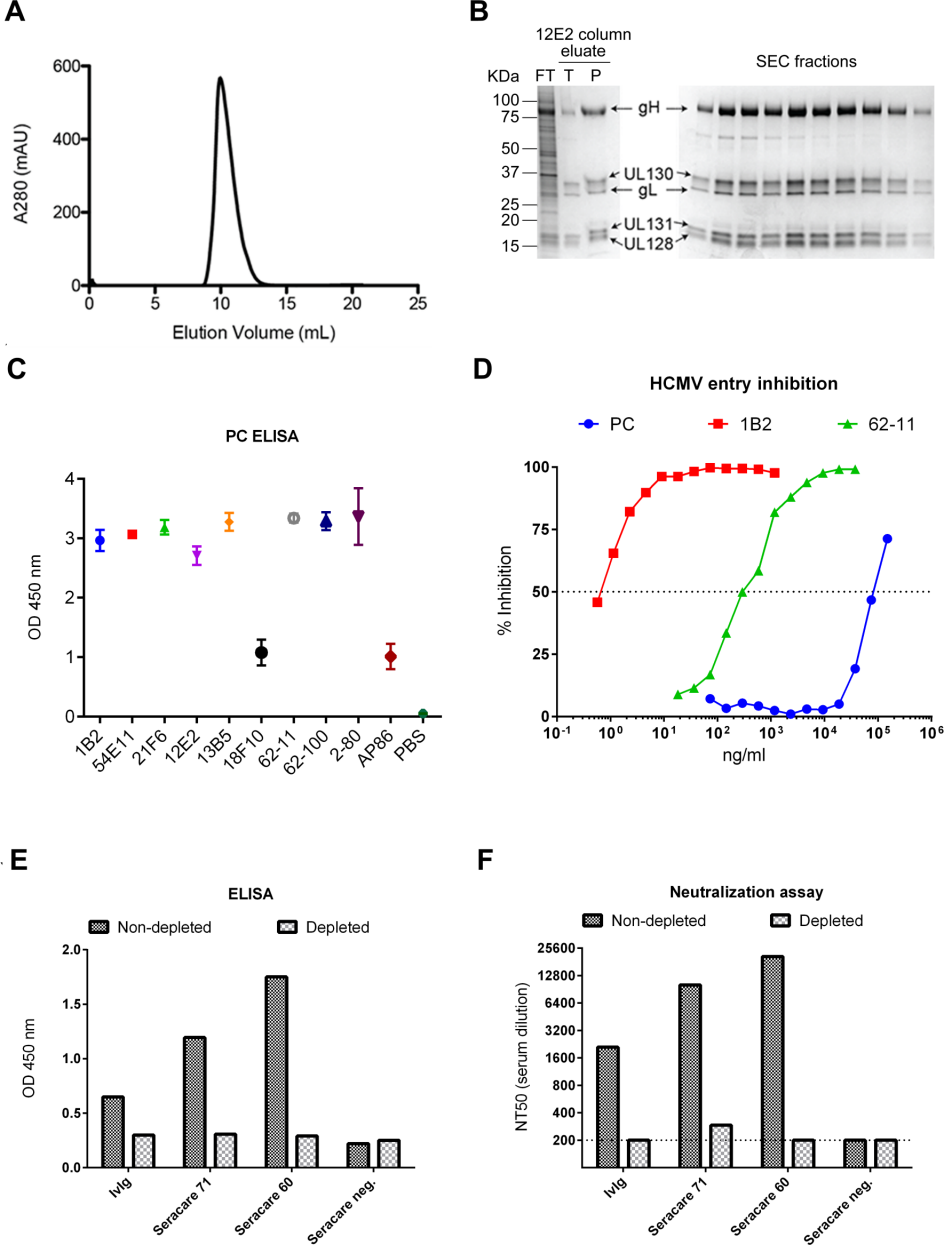
Characterization of soluble PC protein. **A.** Size exclusion chromatography (SEC) on HCMV PC purified from cell supernatant with a 12E2 IgG resin. **B.** SDS-PAGE analysis of PC 12E2 affinity purification eluate and SEC fractions collected in (A) under reducing conditions. kDa = kilo Dalton; FT = flow through, T = test eluate, P = final prep eluate. **C.** ELISA binding of PC- and gH-specific NAb to the purified PC. NAb targeting different epitopes of the UL128/130/131A subunits (1B2, 54E11, 21F6, 13B5) or gH (18F10, 62–11, 62–100, AP86) were tested in saturating amounts to bind the purified PC protein via ELISA. Error bars represent difference in binding of three independent experiments. **D.** Inhibition of EC entry by purified PC protein. Serial dilutions of purified PC protein or, anti-PC NAb 1B2, or anti-gH NAb 62–11 were pre-incubated with HCMV TB40/E and subsequently evaluated via microneutralization assay using ARPE-19 EC to determine the protein/antibody concentration at which 50% HCMV infection was neutralized (NT50). **E and F.** Antibody depletion by purified PC. Commercially available serum products of HCMV seropositive (Seracare 71 and 60) and seronegative (Seracare neg.) individuals or intravenous hyperimmune globulins (IvIg) were serially incubated in ELISA plate wells coated with the purified PC or with mock (1% BSA/PBS). Depleted and mock-depleted samples were tested by ELISA and microneutralization to determine PC-specific binding antibodies (E) and NAb titer (NT50) that block TB40/E infection of ARPE-19 EC (F), respectively.

Using a mammalian expression system optimized for protein production [32] and affinity chromatography utilizing a previously isolated PC-specific NAb [21], we produced monodisperse and highly pure protein complexes that were composed of all five PC subunits (Fig 1A and 1B). SDS-PAGE analysis of the purified protein under denaturing and reducing conditions showed five protein bands that were consistent with previously described molecular weight values of the HCMV PC subunits (~85 KDa for gH; ~35 KDa for gL; ~15 KDa for UL128; ~35 KDa for UL130; and ~18 KDa for UL131A) [33, 34]. ELISA binding using a panel of recently isolated monoclonal NAb provided evidence that the purified protein presented a variety of conformational and linear neutralizing epitopes located within the UL128/130/131A subunits or gH (Fig 1C) [21, 28]. In addition, when the purified protein was used in relatively high concentration compared to two previously isolated NAb targeting epitopes of the UL128/130/131A subunits and gH it effectively interfered with HCMV infection of ARPE-19 EC [21], suggesting that it can saturate receptor binding on these cells, thereby blocking HCMV infection (Fig 1D) [35]. Moreover, the purified protein depleted binding antibodies (Fig 1E) and EC-specific NAb (Fig 1F) in commercially available human HCMV antisera and intravenous hyperimmune globulin preparations (IvIg), indicating that it binds PC-specific antibodies induced by HCMV during natural infection. These results indicated that we produced highly pure soluble PC protein that displayed conformational and linear neutralizing epitopes, interfered with HCMV entry, and was recognized by antibodies induced by HCMV in infected individuals.

### HCMV NAb induction by soluble PC protein in mice

**Fig 2:**
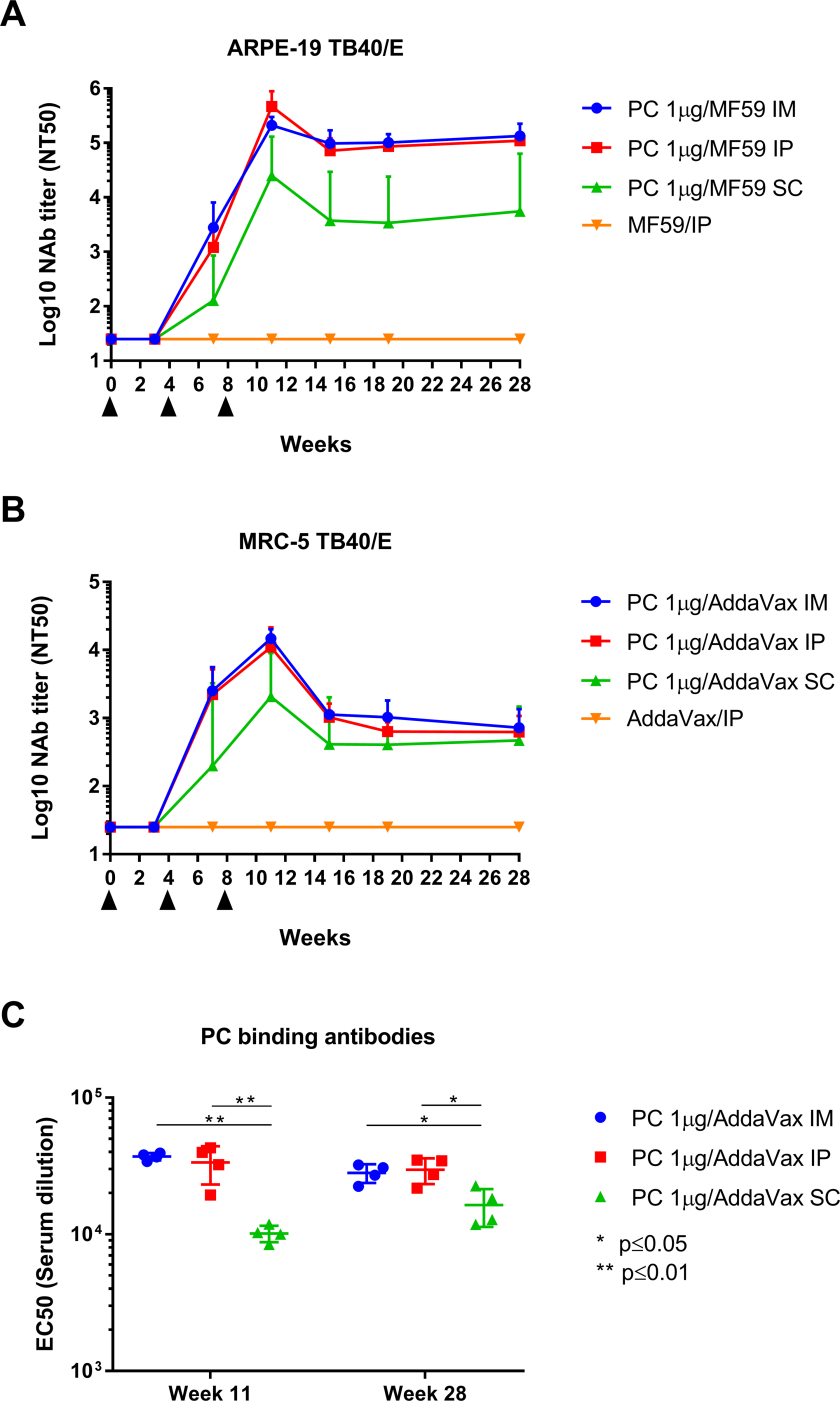
Immunogenicity of purified PC protein to induce NAb and binding antibodies in mice. Balb/c mice (n = 4) were immunized three-times by intramuscular (i.m.), intraperitoneal (i.p.), or subcutaneous (s.c.) route with 1 μg of purified PC protein admixed with AddaVax adjuvant. Control animals (n = 3) were immunized i.p. using AddaVax alone. Serum NAb titers (NT50) were evaluated against TB40/E on ARPE-19 EC **(A)** and MRC-5 FB **(B)** by microneutralization assay at multiple time points over a period of 28 weeks. Black triangles indicate immunizations. Bars represent 95% confidence interval of the geometric mean. PC-specific binding antibody titers (EC50) at week 11 and 28 **(C)** were analyzed by ELISA. Differences between groups were evaluated using multiple t-test (* = p≤0.05, ** = p≤0.01).

In order to determine the immunogenicity of the purified PC protein to stimulate HCMV NAb responses, we evaluated HCMV NAb induction in Balb/c mice following different routes of immunization with the soluble PC protein in combination with an adjuvant (AddaVax) comparable to the clinically approved adjuvant MF59. Balb/c mice were immunized three times with the adjuvanted PC protein by either i.m., i.p., or s.c. route and NAb were measured over a period of 28 weeks by standard microneutralization assay against HCMV strain TB40/E on ARPE-19 EC and MRC-5 FB. As shown in Fig 2, the purified PC protein stimulated robust and durable NAb that blocked HCMV infection of both EC (Fig 2A) and FB (Fig 2B) [22, 25], indicating that a vaccine approach using the PC stimulates NAb targeting the UL128/130/131A subunits that specifically block EC entry, and gH-specific NAb that block both FB and EC entry [21, 22]. While NAb remained undetectable after primary immunization, EC- and FB-specific NAb were detectable after the first boost in all vaccine groups and reached peak levels following the second boost. After reaching peak levels, NAb titer declined, but then remained stable at relatively high levels until the end of the experiment. While EC-specific NAb were generally significantly higher in i.p and i.m vaccinated animals than in s.c immunized animals (p<0.01), FB-specific NAb titer of the different vaccine groups converged over time and were comparable at week 28. Consistent with the difference in EC-specific NAb titer measured between the vaccine groups, binding antibodies in animals immunized with the PC protein via i.p and i.m route exceeded those in animals immunized with the PC protein via s.c route (Fig 2C). As anticipated, mice vaccinated with adjuvant only did not develop measurable HCMV antibody responses. These results demonstrated that the purified soluble PC protein was highly immunogenic in mice to stimulate robust and durable HCMV NAb and binding antibody responses following different routes of immunization.

### Comparison of vaccine strategies to stimulate NAb by the HCMV PC

**Fig 3:**
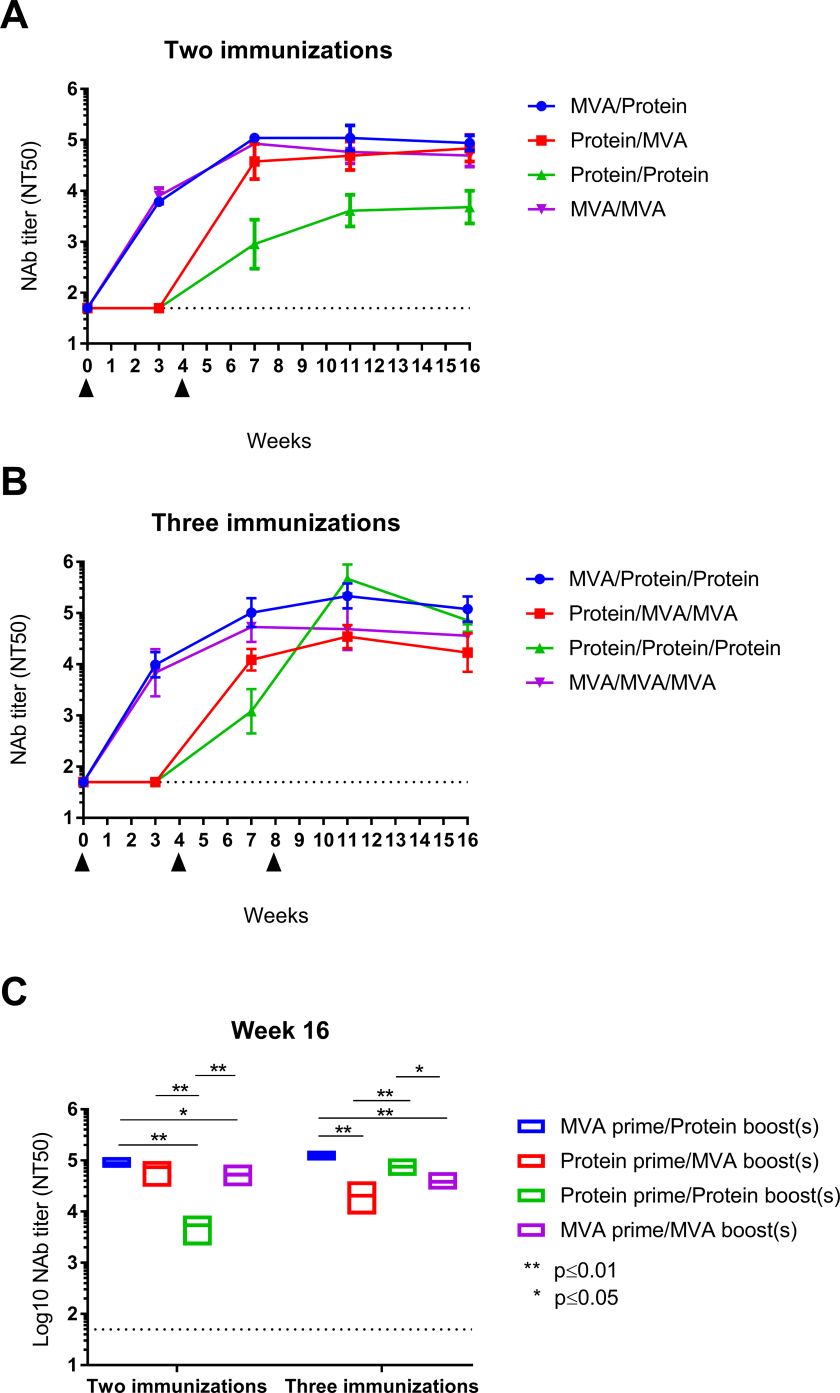
Homologous and heterologous vaccination with MVA-PC and purified PC. BALB/c mice (n = 5) were i.p. immunized with either MVA-PC alone (5x10^7^ PFU), purified PC protein alone (1 μg, admixed with AddaVax), or the indicated prime-boost regimen. Shown are NAb titers (NT50) following two **(A and C)** or three **(B and C)** immunizations that were measured against HCMV TB40/E on ARPE-19 EC. Black triangles indicate immunizations. Bars represent 95% confidence interval of the geometric mean. Floating bars in C represent minimum, maximum, and mean NT50 titers measured on EC at week 16 in the two- and three-immunization regimens. Shown is multiple t-test statistical analysis of NAb titers. (** = p≤0.01, * = p≤0.05).

To compare the potency of soluble PC protein and MVA-PC to elicit HCMV NAb via homologous and heterologous immunization, we evaluated HCMV NAb induction in mice by MVA-PC and adjuvanted PC protein either alone or combined using two and three dose immunization schedules. Balb/c mice were two or three times i.p vaccinated with the different prime-boost combinations and EC-specific NAb were measured against HCMV TB40/E by standard assay over a period of 16 weeks. I.p. immunization was chosen over traditional i.m immunization for consistency with previous immunization studies using MVA-PC in mice [25]. While robust HCMV NAb were induced by homologous and heterologous vaccination using MVA-PC and soluble PC protein, marked differences in the potencies of these vaccine approaches to stimulate HCMV NAb were observed following two or three immunizations (Fig 3). Two immunizations using the MVA-PC vector either alone or combined with soluble PC protein stimulated robust and comparable HCMV NAb responses that significantly exceeded those induced by two immunizations with PC protein alone. In contrast, using a three-dose immunization schedule, NAb induced by soluble PC protein alone and NAb induced by MVA-PC combined with two boosts of PC protein increased to levels of comparable magnitude that exceeded NAb titer stimulated by MVA-PC alone. Overall lowest HCMV NAb titers using a three-dose immunization schedule were elicited by PC protein boosted twice with MVA-PC. Of note, all PC protein immunizations were performed with 1μg of protein based on previous reports using the PC as vaccine antigen in mice [23, 36]. Higher amounts of the PC protein could have potentially enhanced the induction of HCMV NAb [22]. Also, the use of adjuvants other than AddaVax could have led to improved immunogenicity of the PC protein. Yet, previous studies found only minor differences in the potency of a variety of clinically approved adjuvant formulations including MF59 in eliciting HCMV NAb by soluble PC protein [22]. In sum, our results indicated that homologous and heterologous prime-boost vaccination using subunit vaccine approaches based on recombinant protein and MVA stimulates robust NAb by the HCMV PC dependent upon immunization schedule.

## Discussion

By developing a vaccine approach employing soluble HCMV PC protein and utilizing a previously generated MVA vector co-expressing all five HCMV PC subunits (MVA-PC), we compared homologous and heterologous prime-boost vaccine approaches to stimulate NAb by the HCMV PC in mice. Our results suggest that these vaccine approaches confer significantly different potencies to stimulate HCMV NAb dependent upon immunization schedule. While the MVA-PC vector either alone or combined with soluble PC protein is significantly more potent than PC protein alone to elicit robust HCMV NAb by only two immunizations, soluble PC protein either alone or combined with MVA-PC is more potent than MVA-PC alone to elicit HCMV NAb by three immunizations. Cumulatively our results suggest that MVA-PC is sufficient to stimulate robust EC specific NAb by only two immunizations, whereas soluble PC protein alone is sufficient to promote HCMV NAb induction by three immunizations. In contrast to previous observations to enhance antigen specific immunity by prime-boost immunization using MVA and purified protein [37–39], heterologous vaccination using MVA-PC and soluble PC protein does not appear to augment HCMV NAb induction, at least when considering the magnitude of the antibody titers. Whether this immunization approach improves the stimulation of HCMV NAb in a different way for example by expanding the antibody epitope specificity remains to be determined. These results provide first insights into the comparative immunogenicity of MVA and soluble protein to stimulate anti-HCMV PC NAb by homologous and heterologous prime-boost vaccination, which could contribute to develop subunit vaccine approaches for the prevention of HCMV infection.

Several observations made with in vitro studies, clinical samples, and animal models suggest that NAb may play an important role in preventing congenital HCMV infection [11, 12, 21, 40–42]. Intriguingly, a recent study using a novel non-human primate model of congenital rhesus CMV transmission showed that preexisting antibodies with high PC- and gB-specific neutralizing activity can protect against congenital infection following aggressive virus challenge of seronegative pregnant dams [41]. This observation made with an animal model considered to be highly relevant for translational research may suggest that a vaccine candidate able to elicit high-titer and durable NAb could reduce the risk of congenital HCMV infection in seronegative pregnant women. While these studies highlight the potential necessity of antibody neutralizing function in preventing congenital HCMV infection, other antibody functions such as antibody-dependent cell-mediated cytotoxicity or cellular immune responses could contribute to the protection against congenital infection. Future studies should more thoroughly characterize the different functions of antibodies targeting the HCMV PC and investigate whether the PC affords immunogenicity to induce CD4+ or CD8+ T cells. In addition, the analysis of T cells may lead to a better understanding of the impact of modality, regimen, route of immunization, and adjuvant on the immunogenicity of homologous and heterologous prime-boost vaccine approaches using the HCMV PC. Since T cells are the dominant drivers to protect against HCMV disease in stem cell transplant recipients and NAb appear not to contribute significantly to the protection against HCMV infection in these patients [43, 44], a more detailed characterization of the immune responses that target the PC may provide valuable insights into the use of this glycoprotein complex to develop a universal HCMV vaccine candidate.

The significant higher potency of MVA-PC compared to soluble PC protein in stimulating HCMV NAb by only two immunizations may be associated with different PC antigen forms of these vaccine approaches. In contrast to the approach using soluble PC protein, MVA-PC expresses a membrane-associated form of the PC that is tethered via the gH transmebrane domain (TM) to the surface of MVA-PC-infected host cells [25]. This suggests that a membrane-associated form of the PC with gH TM is substantially more immunogenic in eliciting HCMV NAb than a soluble form of the PC without gH TM. Our previous findings for the immunogenicity of MVA-PC in comparison to an MVA vector expressing a soluble form of the PC with deleted gH TM support this conclusion [25]. Membrane-associated PC for example on MVA-PC-infected cells may be displayed in highly ordered or repetitive structures, thereby promoting cross linking of B cell receptors and, hence, thymus-independent B cell activation [45]. In addition, co-stimulatory molecules and antigen cross-presentation by MVA-infected dendritic cells may enhance B cell activation by membrane-tethered PC [46]. Unlike immunization using MVA-PC, vaccination using soluble PC protein is not limited by interference with vector immunity [47, 48], which could explain why soluble PC protein is more potent than MVA-PC in eliciting HCMV NAb by three immunizations. Despite the differences in immunogenicity of MVA-PC and soluble PC protein in stimulating HCMV NAb, the observation that HCMV NAb responses were effectively boosted by combinations of these two vaccine approaches suggest that a membrane-associated form and a soluble form of the HCMV PC have similar antigenic structures.

Patient low compliance with multiple-dose vaccine schedules may compromise vaccine efficacy by reducing the number of patients that complete the recommended immunization series. Missed doses account for approximately two thirds of noncompliance [49], with vaccines requiring more than three doses having the worse compliance rate [50]. Reduced numbers of doses have been shown to improve compliance and consequently lower infection rate and increase cost-effectiveness [51, 52]. For this reason, evidence-based decision making in public health has led to reduced-dose schedules for hepatitis B, pneumococcal, and meningococcal serogroup C vaccine programs [53]. Considering the significant higher potency of MVA-PC compared to soluble PC protein in eliciting NAb responses by only two immunizations, a subunit vaccine approach based on MVA to stimulate NAb by the HCMV PC maybe more compatible with patient compliance than an approach using adjuvanted protein.
